# Loss of Propiconazole and Its Four Stereoisomers from the Water Phase of Two Soil-Water Slurries as Measured by Capillary Electrophoresis

**DOI:** 10.3390/ijerph8083453

**Published:** 2011-08-22

**Authors:** Arthur W. Garrison, Jimmy K. Avants, Rebecca D. Miller

**Affiliations:** 1 Ecosystems Research Division, National Exposure Research Laboratory, U.S. Environmental Protection Agency, 960 College Station Rd., Athens, GA 30605, USA; 2 Senior Service America, U.S. EPA, 960 College Station Rd., Athens, GA 30605, USA; E-Mail: avants.jimmy@epa.gov; 3 Student Services Authority Contract, U.S. EPA, 960 College Station Rd., Athens, GA 30605, USA; E-Mail: rmiller1204@gmail.com

**Keywords:** capillary electrophoresis, propiconazole, stereoselectivity, biotransformation

## Abstract

Propiconazole is a chiral fungicide used in agriculture for control of many fungal diseases on a variety of crops. This use provides opportunities for pollution of soil and, subsequently, groundwater. The rate of loss of propiconazole from the water phase of two different soil-water slurries spiked with the fungicide at 50 mg/L was followed under aerobic conditions over five months; the t_1/2_ was 45 and 51 days for the two soil slurries. To accurately assess environmental and human risk, it is necessary to analyze the separate stereoisomers of chiral pollutants, because it is known that for most such pollutants, both biotransformation and toxicity are likely to be stereoselective. Micellar electrokinetic chromatography (MEKC), the mode of capillary electrophoresis used for analysis of neutral chemicals, was used for analysis of the four propiconazole stereoisomers with time in the water phase of the slurries. MEKC resulted in baseline separation of all stereoisomers, while GC-MS using a chiral column gave only partial separation. The four stereoisomers of propiconazole were lost from the aqueous phase of the slurries at experimentally equivalent rates, *i.e.*, there was very little, if any, stereoselectivity. No loss of propiconazole was observed from the autoclaved controls of either soil, indicating that the loss from active samples was most likely caused by aerobic biotansformation, with a possible contribution by sorption to the non-autoclaved active soils. MEKC is a powerful tool for separation of stereoisomers and can be used to study the fate and transformation kinetics of chiral pesticides in water and soil.

## 1. Introduction

**Figure 1.**
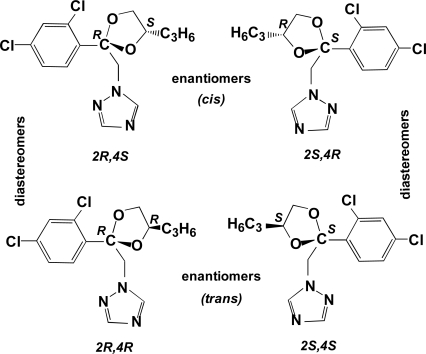
Structures of the four propiconazole stereoisomers.

Propiconazole (Figure 1) is a broad-spectrum 1,2,4-triazole fungicide used for the control of fungal diseases on cereals, bananas, turf, rice, peanuts, stone fruit and maize. It is a systemic foliar fungicide that acts by interfering with ergosterol biosynthesis and inhibition of steroid demethylation [1]. Propiconazole is enzymatically oxidized at the side chain attached to the dioxolane ring and by deketalization with the loss of the dioxolane moiety in plants, soils and fungi.

Since propiconazole is used as an agricultural fungicide, there is concern regarding potential human and wildlife exposure from residues and metabolites in the environment, including soil, sediment, and water receiving soil runoff. Kahle *et al.* [2] detected propiconazole at concentrations of 4–27 ng/L in the influents of all ten wastewater treatment plants examined; its concentration was largely unaffected by wastewater treatment. Kreuger [3], in a study of pesticides in stream water in an agricultural catchment in southern Sweden, found propiconazole at maximum concentrations of 2.8, 20 and 0.6 μg/L at three sites. Propiconazole was one of the pesticides detected in the highest concentrations (130 μg/Kg) in the top 1–2 cm of streambed sediments in 30 Danish lowland streams [4]. A Norwegian study [5] showed that due to its high sorbtivity and mobility, particles of <2 μm can be important carriers of propiconazole in runoff suspensions entering water bodies.

Propiconazole is known to be degraded in soils by hydroxylation of the *n*-propyl side chain and the dioxolane ring as well as with formation of 1,2,4-triazole with DT_50_ (loss of 50% of the parent) values of 40–70 days under aerobic conditions at 25 °C [1]. Another source [6] gives a range of soil half life of 30 to 112 days in an aerobic soil under controlled conditions. In a study of laboratory degradation of propiconazole and three other pesticides in four Norwegian soils, propiconazole was shown to be more persistent than the other pesticides, with persistence depending upon the soil properties [7]. Riise [8], also in Norway, showed that different size fractions of soil possessed different physical and chemical properties and, therefore, different capacities to associate with propiconazole. In laboratory studies on formation and loss of bound residues of propiconazole in soils, Kim *et al.* [9] showed that the half-life of the bound fungicide was about 315 days in sandy loam soil, but was beyond the experimental time limit for silty clay loam soil. Finally, these same investigators showed in another study that most of the propiconazole applied to the soil surface in a rice-paddy-soil lysimeter remained in the top 10 cm of the soil, and a large fraction of that was bound to the organic-rich topsoil [10]).

In addition to possible exposure of these fungicides to humans and wildlife through soil and water residues, their stereoselective transformation to form new and possible more harmful compounds is also of concern. All of the conazole fungicides are chiral, which can be a critical feature in their environmental behavior and toxicity. Many pesticides are chiral and can be metabolized enantioselectively (stereoselectively) by microbes, becoming depleted in one enantiomer while enriched in the other. In addition, the metabolite may be chiral, as is often the case with conazoles [11]. Established data show that a wide variety of chiral pesticides occur non-racemically—existing as unequal concentrations of their enantiomers (or stereoisomers)—in various environmental compartments, are transformed enantioselectively in environmental microcosms, produce chiral metabolites which may themselves be enantioselective, or have enantioselective toxic effects on various organisms [12].

A frequently occurring complication, common to many conazoles, is that many chiral pesticides have more than one chiral center; this results in four or more stereoisomers, each possibly having different biological properties, which leads to stereoselectivity in biotransformation rates, persistence, and toxicity to both target and non-target organisms. This complicates both chemical analysis and risk assessment. For example, since it is known that diastereomer A of the fungicide triadimenol is ten times more acutely toxic to rats (oral LD_50_) than is diastereomer B [13], the stereoselective formation of triadimenol from triadimefon is an important issue for both human health and ecological risk assessment [11].

Propiconazole has two chiral centers, at the 2- and 4-positions of the dioxolane ring, and thus exists as two pairs of diastereomers and two pairs of enantiomers for a total of four stereoisomers (Figure 1). The two pairs of enantiomers are referred to as *cis* [(2R,4S) and (2S,4R)] and *trans* [(2R,4R) and 2S,4S)] [14,15]. The isomers with absolute configuration 2S are more efficient inhibitors of ergosterol biosynthesis than the corresponding 2R isomers. In addition, field trials of the individual stereoisomers showed that each of three different pathogenic fungi species discriminated in its biological activity toward each stereoisomer, and that the fungicidal activity as well as the small plant growth regulating effects of the different isomers varied for different target organisms [14]. In soils, several metabolic products result from hydroxylation of the propyl side chain or of the dioxolane ring. Previous research showed that the metabolism of propiconazole in winter wheat to produce the three side-chain hydroxylation products was stereoselective; *i.e.*, each of the four stereoisomers formed the products at different rates [1,16].

Analysis of the separate stereoisomers of chiral pesticides is usually accomplished by HPLC or GC using chromatography columns with chiral solid phases. Such analysis is also possible using capillary electrophoresis (CE). The analytical approach used here involves the mode of CE known as MEKC (micellar electrokinetic chromatography) [17], which was developed a few decades ago expressly for the CE analysis of neutral analytes. In MEKC, a typical CE buffered electrolyte, such as is used in capillary zone electrophoresis for analysis of ionic analytes, is prepared and a micelle forming compound such as sodium dodecyl sulfate (SDS) for complexing neutral analytes and giving them a charge and resultant mobility in the electrolyte-filled column is added. Finally, for analysis of enantiomers, a chiral selector, e.g., a cyclodextrin, is added to selectively complex the enantiomers of the chiral analyte, creating diastereomers that, in turn, complex with the SDS to different degrees and allow separation by the system. Several articles provide the basic methodology, even recipes, for separation of pesticide enantiomers, including those in environmental samples [17–20].

In this research, a MEKC method was developed for CE separation and analysis of the four stereoisomers of propiconazole and applied to follow the loss of the stereoisomers from the water phase of two different aerobic soil-water slurries over about five months. Gas chromatography-mass spectrometry using a column with a chiral phase was employed as an auxiliary technique. Goals of this research were to demonstrate the application of CE to the analysis of chiral pesticides and their stereoisomers in environmental matrices, as well as to determine whether the loss of propiconazole from soil is stereoselective.

## 2. Experimental

### 2.1. Chemicals

The propiconazole standard was obtained from the U.S. Environmental Protection Agency National Pesticide Standard Repository (Ft. Meade, MD, USA); its purity was 99.6%. Solutions of the fungicide were prepared at 5 μg/mL in methyl *tert*-butyl ether (MTBE) and analyzed by GG-MS to check for purity, which nominally matched the given percentage purity. Methanol, MTBE, other organic solvents, sodium hydroxide and inorganic buffer salts were of analytical grade from Fisher Chemicals (Fair Lawn, NJ, USA). CE reagents, including 2-hydroxypropyl γ-cyclodextrin (the chiral selector) and sodium dodecyl sulfate (for micelle formation) were obtained from Sigma-Aldrich. Reagent water for all experiments was produced by a Barnstead Nanopure Infinity water purification system (Thermo Fisher Scientific, Waltham, MA, USA).

### 2.2. Soil Sources, Collection Data and Physiocochemical Characteristics

**Soil 1 (USDA)**: J. Phil Campbell Natural Resource Conservation Center, Watkinsville, GA, USA. This is a composite agricultural soil collected from the A horizon of a cropped field on this USDA property. After collection, the soil was air-dried, sieved to <2 mm and riffled for homogenization.

**Soil 2 (UGA)**: Univ. of Georgia Horticulture farm, Watkinsville, GA. This is a composited soil collected from the top 10 cm of the peach tree area of the horticulture farm. It was air-dried, sieved to <1 mm and homogenized (this soil was collected at a different time from soil 1 and inadvertently sieved to a smaller particle size.)

*Physicochemical characteristics of soils* [CEC = cation exchange capacity (meq/100g) and OM = organic matter]:

**Soil 1:** pH, 6.2; CEC, 8.9; sand, 70%; soil, 19%; clay, 11%; OM, 3.0%.
**Soil 2:** pH, 6.3; CEC, 13.9; sand, 72%; soil, 18%; clay, 10%; OM, 5.4%.

Soil pH was measured in a slurry of 1:2.5 soil to 0.01 M CaCl_2_ solution (wt:vol). The other soil parameters were measured by a contractor using standard methods.

### 2.3. Propiconazole Exposure to Soils

The experiment required preparation of 50 μg/mL of propiconazole in 1:5 (wt:vol) soil-water slurries of each soil in mineral/nutrient water. For this, 25 mg of propiconazole was dispensed in methanol solution onto the bottom of an autoclaved (121 °C for 20 min) Erlenmeyer flask; the solvent was allowed to evaporate under ambient conditions. Simultaneously, 500 mL of an aqueous solution of minerals and nutrients used to support growth of standard microbial cultures [21] was autoclaved, covered with foil, and cooled. This solution was added to the flask containing the propiconazole and the contents were stirred overnight with a magnetic stirrer at room temperature to produce 50 μg/mL of the fungicide in the mineral/nutrient water, as verified by the GC/MS analytical method described below. Aliquots of 20 mL of this aqueous solution were transferred to each of several 50 mL amber serum vials which had been autoclaved. Four grams of soil 1 (not autoclaved) were then added to each of these vials. This process was repeated with soil 2. These vials were then stoppered and shaken overnight, and constituted the active (spiked) treatments.

There were also produced, by autoclaving the soils, a corresponding number of controls. For this, 4 g of soil 1 was wetted with 2.0 mL of water and transferred to each of several amber serum vials; this was repeated for soil 2. These soils were autoclaved (121 °C for 20 min) once a day for 3 days; then 20 mL of the above autoclaved mineral/nutrient solution containing 50 ug/mL of propiconazole was added to each vial and the vials were shaken overnight. All vials, of both active and control treatments, for these kinetic experiments were shaken continuously in the dark in a thermostated shaker at 20 °C for the course of the experiment.

### 2.4. Extraction and Sample Preparation Methods

At t_0_ and each successive time point, aliquots of the water phase were taken from all vials, active and control, for CE and, in some cases, GC-MS analysis. First, each vial was manually shaken to mix the slurry, then the stopper was immediately removed and about 1mL of the slurry was collected with a wide-mouth pipet and dispensed into a 2 mL centrifuge tube. The tube and contents were centrifuged at 14,000 rpm for 20 min (Eppendorf centrifuge 5418). As much as possible of the aqueous phase was removed with an automatic pipet. For CE analysis, 40 μL of this was fitered through a 0.45 μm syringe filter into a CE analysis vial. For GC-MS, 0.5 mL of this phase was extracted with 0.5 mL of methyl *tert*-butyl ether (MTBE) by vibration with a vortex mixer; 100 μL of this extract was diluted with 900 μL of MTBE for final GC-MS analysis.

### 2.5. Propiconazole Recovery Data

Recovery from deionized water spiked at 50 μg/mL; average of duplicates: 97%.

Recovery from spiked soil-water slurries: a slurry of 5 mL of water and 0.5 g of soil 1 containing 50 μg/mL propiconazole was prepared as described above (section 2.3) and shaken overnight at 24 °C. The slurry was vortexed, a 1 mL sample of the slurry was collected, and the water was extracted as described above (see Extraction and Sample Preparation Methods) for subsequent analysis by CE. The solid (soil phase) remaining in the centrifuge tube was washed with 1.0 mL water, which was discarded. (This step was included to remove most of the soil pore water, which may have contained dissolved propiconazole, from the soil phase to allow for more accurate soil analysis.) The wet soil was extracted by vortexing with 1.0 mL of MeOH 2 times; extracts were then combined and evaporated to 0.5 mL. MeOH was selected as the extraction solvent because of its water miscibility. This extract was diluted with 1.5 mL of water to achieve an appropriate 25% MeOH aqueous solution for CE analysis; 40 μL of this was filtered through a 0.45 μm syringe filter. Both water and soil phases were analyzed by CE (see section 2.6). This procedure was repeated for soil 2, in duplicate.

Average % recovery (duplicates) of propiconazole from water phase of spiked soil:water slurry: Soil 1, 34.7%; Soil 2, 30.9%.

Average % recovery (duplicates) of propiconazole from soil phase of spiked soil:water slurry: Soil 1, 40.6%; Soil 2, 34.1%.

Total recoveries (from water + soil): Soil 1, 75.3%; Soil 2, 65.0%. Apparently, about 25% and 35%, respectively, of propiconazole was irreversibly sorbed to the soil, lost in the 1 mL of water used to wash the soil, or lost by sorption to glassware during the extraction process. The total recoveries are considered adequate for these experiments.

### 2.6. CE Analysis

CE analysis was with a liquid cooled Beckman P/ACE System 5500 CE, with diode array UV detector, hydrodynamic injection, power supply up to 30 kV, and System Gold version 8.1 chromatography software (Beckman Instruments, Fullerton, CA, USA). The CE column was uncoated fused silica: 75 μm id, 300 μm od, 57 cm total length, and 50 cm effective length (MicroSolv Technology Corp., Long Branch, NJ, USA). Samples were contained in vial inserts for small volumes (40 μL or 400 μL, Beckman Instruments). The CE samples prepared during the sample extraction procedure described above were filtered through a 0.45 μm nylon syringe filter (or equivalent) just before transfer to the CE vials.

Before analysis of each sample, the column was washed with distilled water for 2 min, 0.10 M NaOH for 2 min, water again for 2 min, and electrolyte solution for 2 min. The column was maintained at 23 °C. Sample injection was hydrodynamic, usually for 6.5 sec; injections of 10 sec were used if more sensitivity was needed. Typical MEKC electrolyte composition was: 25 mM NaPO4 buffer, pH 7; 75 mM sodium dodecyl sulfate; 30 mM 2-hydroxypropyl-γ-cyclodextrin; and 10% methanol and 5% acetonitrile as organic modifiers. Instrumental conditions: temperature, 23 °C; detector wavelength, 190 nm; voltage, 30 kV; and run time, 30 min. The limit of quantitation for each of the 2 minor propiconazole stereoisomers in the water phase of a water-soil slurry was observed to be about 0.3 mg/L. However, for quantitation of all four stereoisomers (total propiconazole), the limit is about 2.5 mg/L.

### 2.7. GC-MS Analysis

GC-MS analysis of propiconazole standards and extracts was by use of a Hewlett-Packard 5973 mass spectrometer linked to a 6890 gas chromatograph equipped with a BGB 172 (BGB Analytik AG, Switzerland) chiral column. Column description: 30 m × 0.25 mm ID × 0.25 μm film thickness; chiral stationary phase, 20% *tert*-butyldimethylsilylated-β-cyclodextrin. GC conditions were: injection, splitless at temp. 275 °C; column temp. program, 150–220 °C at 4°/min, followed by temp. hold for 60 min; helium gas flow, 1.5 mL/min; MS inlet temp., 275°; MS source temp., 230°; and fragmentation voltage, 70 eV. Sample injection volume was 1 μL. Detection was by selected ion monitoring (SIM); SIM ions were *m/z* 173, 259 and 261 for propiconazole.

Quantitation for both CE and GC-MS was by comparison of stereoisomer peak areas to those of propiconazole standards of similar concentration analyzed the same day; quality control included analysis of at least one standard per day as well as analysis of a standard before and after each 10 samples. These standards were in turn referenced to a standard curve of propiconazole stereoisomer peak area *vs.* concentration. For example, a standard curve was generated by CE using a series of 3, 5, 8, 10, 50 and 100 mg/L standards of propiconazole in water plotted against CE peak area to give a linear plot with R^2^ of 0.9922. Reproducibility of propiconazole quantitation by CE was measured using a 5 mg/L standard solution in water, with n = 6. The percent relative standard deviation for peak 1 stereoisomer was 5.6%, and for peak 2, 7.3%. The RSD for the 2 minor peaks were higher because of their low concentrations (about 0.5 mg/L each): peak 3 was 31.9% and peak 4 was 36.1%. The RSD for total propiconazole was 18.9%.

## 3. Results and Discussion

### 3.1. CE Data and Stereoisomer Resolution

**Figure 2.**
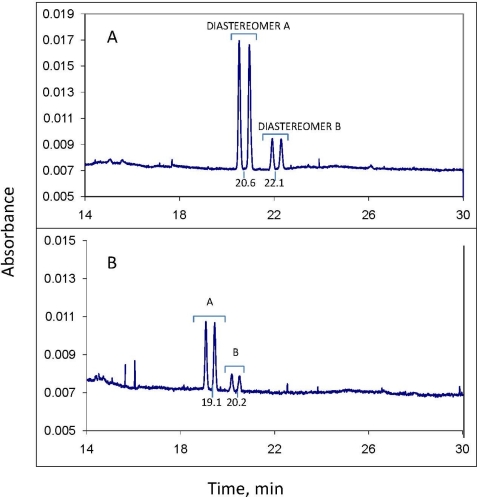
(**A**) CE electropherogram of sample prepared from the aqueous phase of soil 1 soil-water slurry after exposure of the slurry to 50 mg/L propiconazole for 2 hours; concentration of propiconazole in aqueous phase, 9.8 mg/L. Numbers below baseline are the migration times of the two diastereomers. See Experimental section for CE experimental details. (**B**) Electropherogram of aqueous phase of soil 1 sample after exposure for 96 days; concentration of propiconazole in aqueous phase, 3.8 mg/L. Notice shift in migration times relative to the 2 hour sample.

Propiconazole consists of four stereoisomers (Figure 1) [1,14,15]. The separate stereoisomers were not available to us, so we were not able to assign absolute configurations or signs of optical rotation to the individual peaks in the electrophorogram [Figure 2(A)]. It is assumed however, from other work [11], that the two sets of peaks with identical enantiomer concentrations within each set indicate the two diastereomers of propiconazole (actually, this term is used loosely—as shown in Figure 1, the pairs of diastereomers are divided between the two sets of enantiomers). It is also assumed in this work that diastereomer A is the larger of the two, since one report of the synthesis of propiconazole states that diastereomer A constitutes 60% of the product while diastereomer B is 40% [22]. The proportions of our standard propiconazole are different, however; diastereomer A is 81% while B is 19% [Figure 2(A)]. Regardless, it is assumed here that the differences in proportions of diastereomer A *vs.* B results from the synthesis process and not from any differences in UV absorbance. This is verified by the fact that the ratios of diastereomers as measured by CE with UV detection are very similar to those measured by GC-MS (see DF a/b, Table 1).

**Figure 3.**
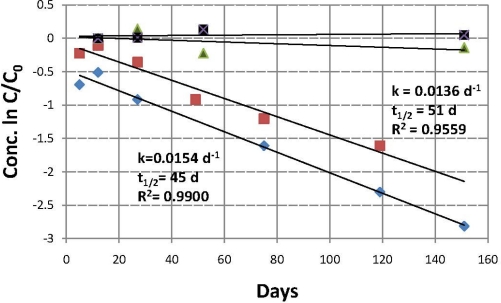
First order plots for loss of propiconazole from the water phases of soils 1 and 2 soil:water slurries and corresponding controls. Live samples and controls were spiked at t_0_ with 50 mg/L propiconazole. Plots begin with day 5 data to allow for initial loss by sorption. Controls show little, if any, loss of propiconazole. Legend: 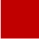 soil 1, active; 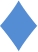 soil 2, active; ▪ soil 1, control; 
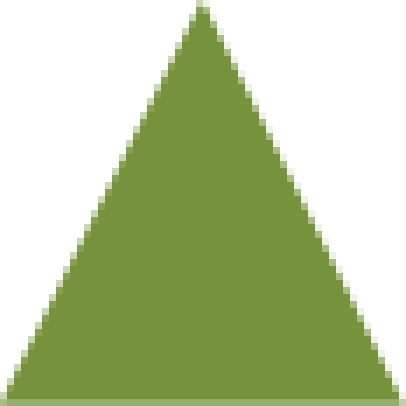
 soil 2, control.

Figure 2(A) is the CE electropherogram of a sample prepared from the aqueous phase of the soil 1 soil-water slurry after exposure of propiconazole to the slurry for 2 hours. The slurry had been spiked at t_0_ with 50 mg/L of standard propiconazole. This standard gave an identical electropherogram pattern to that of Figure 2(A). The concentration of total propiconazole at 2 hours in the water phase of soil 1 was 9.8 mg/L, as measured by CE. The 2 hour samples were the t_0_ samples for subsequent kinetic runs; it would have been difficult to obtain and process samples for analysis in a shorter time. Apparently the majority of the fungicide had sorbed to the soil by 2 hours (see section 2.5). The kinetic data in Figure 3 show that the t_1/2_ of propiconazole loss in both soils is on the order of 50 days under the conditions used here, so only a small amount would have been lost by chemical reaction in 2 hours. Figure 2(B) is the electropherogram of an aqueous-phase sample after exposure of the soil 1 slurry for 96 days; the concentration of total propiconazole is 3.8 mg/L.

The reproducibility of CE migration times is sometimes problematic. Although stable electropherograms and baseline separation of the enantiomers are seen in both Figure 2(A) and 2(B), it is also seen that the migration time of propiconazole in 2(B) relative to 2(A) has decreased from about 20.6 min to 19.1 min for diastereomer A and a corresponding amount for diastereomer B. Slight changes in buffer composition from day to day or in capillary column surface character, which can happen as the column ages, affects migration times by changing the degree of protonation of the silica column surface. Such changes occurred even with propiconazole standard analytes, and sometimes even during the same day. This is not critical for experiments such as described here; since the stereochemical pattern of the propiconazole is apparent (and would be even after some stereoselective biotransformation) the analyte is easily identified, even with some migration time change.

### 3.2. GC-MS Data

GC-MS was used occasionally as an adjunct or confirmatory analytical tool for chiral analysis of propiconazole. GC-MS chromatograms of a propiconazole standard, injected concentration of 5 μg/mL, were generated using a BGB-172 chiral column. The two diastereomers were separated by 4.7 min; enantiomer separation averaged 0.21 min (13% separation) for the first eluting pair and 1.16 min (baseline separation) for the second pair. Similar diastereomer concentrations were obtained by GC-MS as by CE: A constitutes 80%, while B is 20% of the propiconazole mixture (Table 1). The enantiomer peaks for each of the diastereomeric pairs were nominally of equal area, indicating the expected racemic mixtures; the EF (enantiomer fraction) of the first set of enantiomers was 0.48, while that of the second set was 0.50. The lack of a racemic value (0.50) for the first pair of enantiomers is because of poor chromatographic separation of the two peaks; the average length of peak separations at peak apexes relative to total peak heights was only 13%. No other columns were tried for better stereoisomer separation—there possibly are better columns for this purpose than the BGB-172. Contrary to these results, excellent separation of all four stereoisomers of triadimenol, a triazole fungicide similar in structure to propiconazole, were obtained on the same BGB-172 column in earlier work of the authors [11]. The most obvious reason for this discrepancy is that triadimenol and propiconazole have enough differences in structure—e.g., the former compound has a secondary OH and a *tert*-butyl group connected to a carbon chiral center at the same relative position where propiconazole has a substituted dioxolane ring with two carbon chiral centers—to affect their interactions with the *tert*-butyldimethylsilylated-β-cyclodextrin chiral phase of the BGB 172 column.

### 3.3. Loss of Propiconazole from the Water Phase of the Two Soil Slurries

The main objective of this research was to demonstrate the capability of CE to separate and quantify stereoisomers of conazole fungicides and similar chiral compounds in environmental water and soil samples. Therefore, an application to spiked environmental matrices was attempted.

To measure loss of propiconazole from soil slurries, each of two soils was shaken in an aerobic slurry of 1:5 soil-water for about five months; the water had been spiked with 50 μg/mL of propiconazole. Although several products are reported to be formed by biotransformation or abiotic degradation of propiconazole in soils [1], no reaction products were detected in any of the soil or water samples analyzed here. However, our CE method relied only on UV detection and the particular CE conditions used for propiconazole stereoisomer separation and would possibly not have detected any products. The GC-MS method used the SIM mode of detection, which would not have detected products unless they contained the specific SIM ions used for propiconazole. Therefore, there could have been unobserved products of either an abiotic or biotic reaction.

Figure 3 shows plots for the first order rate equations for loss of propiconazole from the water phases of soils 1 and 2. Each time point represents only one CE analysis. The plots begin with day 5 data, assuming that most sorption had occurred by that time. It is seen that the rates of loss are very similar; k values are 0.0136 and 0.0154 d^−1^ for soils 1 and 2 respectively, with half lives of 51 and 45 days. This compares well with the DT_50_ ranges of 40–70 days [1] and 30–112 days [6] obtained by earlier investigators for loss of propiconazole under aerobic conditions. The close k values are not surprising because the properties of the two soils are quite similar (see section 2.2); the pH values are 6.2 and 6.3, while the percent organic matter is 3.0 and 5.4 and the percent clay is 11 and 10.

Analysis of the controls (data shown in Figure 3), which had been autoclaved to reduce or eliminate microbial activity and resulting biotransformation of propiconazole, showed that very little, if any, propiconazole was lost from the soil 1 and 2 water phases during the experiment. This indicates only a very small amount of microbial activity in the controls. Therefore it is concluded that the disappearance of propiconazole from the active samples was probably caused by aerobic biotransformation. However, it is known that autoclaving can cause changes in the character of soil organic matter, and such changes might influence soil sorbtivity. Because the controls were autoclaved and the active samples were not, there could have been some sorption by the active samples and not by the controls. So only abiotic transformation can be excluded from pathways of propiconazole loss in the soils studied here.

### 3.4. Stereoselectivity During Propiconazole Loss

For the 2 hour and 96 day exposure samples of soils 1 [Figure 2(A) and 2(B], respectively] and 2, the enantiomer fractions (EF A and EF B) of each pair of propiconazole enantiomers and the diastereomer fractions (DF) of diastereomers A and B are shown in Table 1.

The EF and DF are calculated according to the following equations, where brackets indicate concentration:

EF=[1st eluting enantiomer]/[1st eluting enantiomer+2nd eluting enantiomer]

DF=[1st eluting diastereomer]/[1st eluting diastereomer+2nd eluting diastereomer]

It is seen from the table that the EF of both pairs of enantiomers of soil 1 changed very little during these 96 days, but the DF tended to decrease (statistics could not be calculated because there were not replicate data points). For soil 2, the EF of both pairs of enantiomers tended to decrease, as well as the DF. These trends indicate a low level of stereoselectivity between both pairs of enantiomers and the diastereomers during the biotransformation of propiconazole in soil 2, and a small degree of diastereomer selectivity for soil 1. Contrary to these low levels, the enantiomer fraction of the conazole fungicide triadimefon was shown to change from about 0.45 to 0.60 in its biotransformation to triadimenol, indicating a higher degree of stereoselectivity [11].

The reproducibility of EF measurements was tested by multiple runs of a 10 mg/L standard of propiconazole. For CE analysis of enantiomers 1 and 2, the EF (EF A in Table 1) was 0.50 ± 0.03 (n = 5), while the EF for enantiomers 3 and 4 (EF B) was 0.49 ± 0.03 (n = 5). EF and DF values for GC-MS analysis of propiconazole stereoisomers are also provided in Table 1 for comparison with the CE data, and are seen to be similar.

## 4. Summary and Conclusions

### 4.1. Capillary Electrophoresis and Its Applications to Environmental Samples

**Figure 4.**
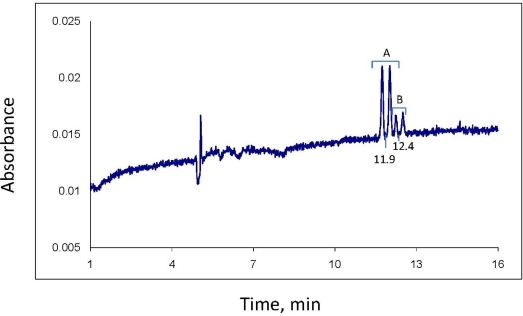
Electropherogram of sample prepared from the soil phase of soil 1 soil:water slurry after exposure of slurry to 50 mg/L of propiconazole for about 1 day; concentration of propiconazole in soil, 36.8 mg/Kg. Numbers below baseline are the migration times of the two diastereomers.

As evidenced by Figure 2, CE is capable of excellent enantiomer separation with minimum background noise and reasonable sensitivity. The level of quantitation for propiconazole under the conditions used here is about 0.3 mg/L for each of the minor stereoisomers in the injected sample and about 2.5 mg/L for total propiconazole, which includes quantitation of all four stereoisomers. One distinct advantage of CE for analysis of environmental water and soil samples, in addition to high resolution and the small volume of sample needed, is the minimum sample preparation required. For the type of sample involved here, the water sample can be injected directly after filtration through a syringe filter. To increase sensitivity in the water phase, one needs only to perform a simple liquid-liquid extraction of a larger amount of water sample, evaporate the organic extraction solvent to a small volume, say 250 μL, and dilute this to 1 mL with water (CE can accommodate such solutions of water and soluble extraction solvents). For solid phase samples such as soil or sediment, conventional extractions with methanol or acetonitrile, for example, can be adapted for CE (Figure 4). The organic extract is simply diluted with water and filtered.

Thus, CE, especially in the MEKC mode, is a useful analytical tool for measuring the kinetics of disappearance/biotransformation of stereoisomers of chiral pesticides and other pollutants from soil, sediment or aquatic microcosms spiked with 10 to 50 mg/L or so of the pollutant (this much may be necessary to follow the concentration as it decreases to low mg/L levels) [18,19], and could be used to simply detect propiconazole or similar pollutants in water or soil at about 1–2 mg/L. In fact, recently developed on-line preconcentration methods coupled with MEKC may allow increases in sensitivity for pesticide detection by up to 100-fold [20].

In addition to its inherent low sensitivity, which can be improved with equipment and technique changes, a disadvantage of CE for trace pollutant analysis is, in some cases, low migration time reproducibility. This can confuse electropherogram interpretation and affect peak identity. As discussed above, changes in buffer/electrolyte composition and/or CE column surface protonation can change migration times on a day-by-day basis. However, an intentional change of the fused silica column may create large migration time changes. The new column used for soil phase analysis (Figure 4) resulted in reduction of time of the propiconazole diastereomer A from 20.6 [Figure 2(A)] to 11.9 minutes (Figure 4). The column was likely not well conditioned, and the change from an aqueous (water phase) to a soil phase extract probably introduced critical salt content and other changes in the injected sample.

### 4.2. Loss of Propiconazole from Soil:Water Slurries

Kinetic data developed in this study using CE showed that soils 1 and 2, which have similar physiochemical properties, support reaction with propiconazole in soil-water slurries to reduce its concentration in the overlying water phase by about two-thirds over a period of three months (Figure 2). The very low loss of propiconazole from the water phase of the autoclaved control samples argues against loss from the active samples by abiotic processes; aerobic biotransformation, however, is a possible and perhaps the likely process for depletion of propiconazole in the environmental systems studied here. The low level of stereoselectivity observed during biotransformation is unusual but, as with the case of imazaquin in aerobic soil slurries [19] and metalaxyl in aerobic soils at pH 4–5 [23], is not unknown.

There remains the possibility of propiconazole loss by a long-term and log-linear sorption process, and the lack of pronounced stereoselectivity supports this because it is believed that sorption of chiral organics to soils and sediments is not stereoselective except in the cases of pure mineral sorbents or definite mineral surfaces [24]. However, diastereomers, as opposed to enantiomers, differ in their abiotic chemical and physical properties. Thus, the observation of slight decreases of DF values in both soils at 96 days exposure (Table 1) could be caused by diastereomer-selective sorption, as was observed by other workers with the higher sorption affinity of 17β-estradiol over that of 17α-estradiol [25].

### 4.3. Risk Implications

As with other chiral pesticides, the stereoisomers of propiconazole are independent entities in respect to their biological properties. Stereoisomers may differ in their toxicities to a variety of species, and may be transformed by microbes at different rates, even with different products. These observations should be brought to bear when the exposure and toxicity of chiral pesticides are being measured or considered in environmental matrices [26]. The effective exposure and toxicity of a chiral pesticide depends upon its stereochemical composition at the point of exposure; furthermore, because of stereoselective biotransformation, this composition may well differ from that of standards to which comparison may be attempted [11].

## Figures and Tables

**Table 1.** Enantiomer and diastereomer fractions (EF and DF) for propiconazole standards using CE and GC-MS and for propiconazole in soils at 2 hours and 96 days using CE.

	**INSTRUMENT**		**SOIL 1**		**SOIL 2**	

	**GC-MS** (n = 3)	**CE**(n = 5)	**2 hours**	**96 days**	**2 hours**	**96 days**
**EFa**	0.48 ± 0.02	0.50 ± 0.03	0.51	0.51	0.51	0.48
**EFb**	0.50 ± 0.00	0.49 ± 0.03	0.50	0.51	0.52	0.46
**DF a/b**	0.80 ± 0.01	0.81 ± 0.01	0.81	0.78	0.80	0.77
